# Clinical Thyrotoxicosis Resulting from Liothyronine Augmentation of Antidepressant Therapy in an Adolescent

**DOI:** 10.1155/2022/2270202

**Published:** 2022-05-24

**Authors:** Benjamin A. Pallant, Claire E. Moore, Lisa Swartz Topor

**Affiliations:** ^1^Warren Alpert Medical School of Brown University, Providence, RI, USA; ^2^Department of Pediatrics, Warren Alpert Medical School of Brown University, Providence, RI, USA; ^3^Hasbro Children's Hospital, Providence, Rhode Island, USA; ^4^Division of Pediatric Endocrinology and Diabetes, Hasbro Children's Hospital, Providence, Rhode Island, USA

## Abstract

*Background/Objective*. Thyrotoxicosis, a condition resulting from excessive peripheral thyroid hormone, is typically accompanied by thyroid function tests demonstrating a high free thyroxine (free T4) with appropriate suppression of thyroid-stimulating hormone (TSH). *Case report*. We describe a 17-year-old female presenting with symptoms of thyrotoxicosis along with suppressed TSH and low free T4, a laboratory pattern concerning for central hypothyroidism. Further history revealed that she was prescribed liothyronine as an adjunct therapy for depression. *Discussion*. Due to the short half-life of liothyronine, clinical signs and symptoms of thyrotoxicosis may develop before detection by interval lab monitoring. *Conclusion*. This case highlights the need for close monitoring and caution when treating adolescents with liothyronine and the importance of interpreting atypical laboratory findings within clinical context.

## 1. Background

Thyrotoxicosis occurs when inappropriately elevated levels of circulating thyroid hormones lead to increased thyroid hormone action [1]. Signs and symptoms of thyrotoxicosis include unintended weight loss, heat intolerance, insomnia, increased frequency of bowel movements, tremor, anxiety, palpitations, atrial fibrillation, and muscle weakness [1]. Either overproduction of thyroid hormone or increased release of preformed thyroid hormone following destruction of thyroid follicles can cause thyrotoxicosis. Common causes of the former include Graves' disease, toxic multinodular goiter, and toxic adenoma [1]. Subclinical thyroiditis and early autoimmune thyroiditis are examples of the latter. Excess exogenous administration of thyroid hormone(s), either to treat hypothyroidism or for other uses, can also cause symptoms of thyrotoxicosis. Thyrotoxicosis is typically characterized by elevated thyroxine (T4), free thyroxine (free T4), and/or triiodothyronine (T3) with appropriately suppressed thyroid-stimulating hormone (TSH). When laboratory findings in the setting of suspected thyrotoxicosis are not consistent with these usual patterns, additional information about the patient's history may be useful to identify the cause of thyroid-hormone abnormalities.

Treatment-resistant depression and the role of thyroid hormone augmentation have been explored in severe major depressive disorder (MDD) that does not response to initial treatment. While SSRIs can be effective in up to a third of patients with MDD, the majority of patients require alternate or additional medications to achieve response, and up to 40% of patients will have continued symptoms after two antidepressant trials [2]. Medications for treatment-resistant depression, such as serotonin norepinephrine reuptake inhibitors (SNRIs), tricyclic antidepressants (TCA), monoamine oxidase inhibitors, mirtazapine, adjunctive atypical antipsychotics, adjunctive lithium, and thyroid hormones have been investigated in adults as either alternative or adjunct therapies. Nonpharmacologic interventions include electroconvulsive therapy and repetitive transcranial magnetic stimulation [3]. The evidence for liothyronine is strongest when used as an adjunct to TCA therapy [4]. Evidence for liothyronine as an adjunct to other antidepressant therapies, including SSRIs, is more limited and has shown mixed results [3, 5, 6]. Little has been reported on liothyronine as an adjunct therapy to antidepressants in youth with depression.

The primary aim of this report is to describe a case of thyrotoxicosis due to administration of liothyronine as an adjunct for treatment of depression in a 17-year-old female without underlying thyroid dysfunction. Her initial laboratory findings were concerning for central hypothyroidism with low TSH and free T4; however, her symptoms aligned more closely with thyrotoxicosis and warranted further investigation. We will also review the uses of liothyronine in the treatment of thyroid disease and psychiatric disease.

## 2. Case Report

A 17-year-old female presented to her pediatrician via telemedicine due to two to three months of lightheadedness, intermittent upper- and lower-extremity paresthesias, insomnia, and occasional twitching. The lightheadedness occurred two to three times each week, was associated with nausea, and resolved with sitting down. These symptoms began a few weeks after a change in her medication regimen for depression. At that time, escitalopram, the initial medication she had received for treatment of depression, had been discontinued, and venlafaxine and liothyronine were started. Liothyronine, initially prescribed as 25 *μ*g daily, was subsequently increased to 50 *μ*g daily. She denied taking any additional medications, vitamins, or supplements. Following the visit with her pediatrician, laboratory studies were notable for low TSH of 0.02 *μ*IU/mL and low free T4 (Table 1), and she was referred to pediatric endocrinology.

At the pediatric endocrinology evaluation, the patient had a normal pulse of 85 beats per minute and elevated blood pressure of 127/75 mm Hg. Her body mass index (BMI) was 18.1 kg/m2, unchanged over the past nine months. Thyroid examination was normal. A total T3 level was obtained and was elevated (Table 1).

Due to both the timeline of symptom onset in relation to the liothyronine treatment and the pattern of suppressed TSH with elevated total T3, the patient was advised to discontinue the liothyronine with close monitoring of psychiatric symptoms by her psychiatrist. She reported complete resolution of lightheadedness, twitching, and insomnia within two weeks of liothyronine discontinuation, and repeat laboratory testing three months later showed normalization of thyroid function (Table 1).

## 3. Discussion

This case demonstrates the need to interpret thyroid hormone laboratory patterns, especially atypical ones, in the context of a patient's history and clinical symptoms. TSH suppression is an extremely sensitive indicator of elevation in circulating thyroid hormones. Laboratory findings in thyrotoxicosis are typically characterized by appropriately suppressed TSH in response to elevated T4 coming from the thyroid, ingestion of exogenous levothyroxine, or rarely an ectopic source such as a neoplasm. Low TSH can also be seen in central hypothyroidism, which was an initial concern in this case. With central hypothyroidism, insufficient production of TSH due to hypothalamic or pituitary dysfunction is the primary abnormality. Inappropriately normal TSH with low free T4 is most consistent with hypothalamic dysfunction while low TSH with low free T4 is rare and suggests a primary pituitary abnormality. In practice, laboratory patterns of hypothalamic and pituitary dysfunction can overlap [7]. Central hypothyroidism, whether hypothalamic or pituitary in origin, can present with symptoms similar to those of hypothyroidism due to other etiologies with the exception of goiter, which is only seen in primary hypothyroidism.

For the patient described in this report, the laboratory findings and the clinical presentation initially appeared to contradict. The pattern of low TSH and low free T4 levels raised concern for central hypothyroidism, but the symptoms were consistent with thyrotoxicosis. The added historical information of recent liothyronine treatment and dose titration offered a unifying explanation of iatrogenic thyroid-hormone excess. Similar patterns of thyroid studies with suppressed TSH and elevated total T3 have been described due to weight loss supplements containing liothyronine [8] or consumption of meat products containing animal thyroid [9]. Isolated elevations in T3 have also been reported in patients with poorly differentiated thyroid carcinoma with coexisting Graves' disease [10], struma ovarii, autoimmune thyroiditis, and Graves' disease with isolated T3-toxicosis [11]. In these various cases, presentations have ranged from mild to severe thyrotoxicosis symptoms.

While liothyronine has been explored as a treatment for thyroid disease, the effects of liothyronine have not been well studied in adolescents or children. The Pediatric Endocrine Society recommends monotherapy with levothyroxine for acquired hypothyroidism in youth [12]. In specific cases of congenital hypothyroidism with central resistance to thyroid hormone, the addition of liothyronine to traditional levothyroxine therapy has been reported to facilitate achievement of a biochemically euthyroid state [13].

Liothyronine has also been studied in adults as an adjunct to augment the effects of SSRIs and TCAs for treatment-resistant unipolar depression [6, 14, 15]. Studies present conflicting data regarding the added benefit of liothyronine for adults [5, 6]. In some studies, the addition of adjunct liothyronine had superior response rates (at least 50% improvement) on the Hamilton Rating Scale for Depression (HRSD) when compared to monotherapy with SSRIs or TCAs [6, 14]. In the STAR∗D study, rates of remission of treatment-resistant depression did not differ significantly with the addition of either liothyronine or lithium to existing regimens [16]. Other studies did not show benefit with adjunct liothyronine [15]. The American Thyroid Association reports that there is low-quality evidence for use of liothyronine in euthyroid individuals with depression [17]. The combination of liothyronine with a SNRI, the regimen prescribed for the patient described in this case, has not been well-studied [6].

Among pediatric and adolescent populations, studies of liothyronine for psychiatric indications are insufficient to remark on either benefit or safety. The only available study of liothyronine in euthyroid children was published in 1973 and described the effects of liothyronine in 20 young children with severe psychiatric disturbance. Doses were titrated as tolerated to a maximum of 75 mcg daily. Thirteen of the patients showed marked improvement in psychiatric features, while two had worsening of symptoms. Common side effects included tachycardia and diarrhea, and those treated with the highest doses experienced weight loss, worsened psychosis, irritability, and insomnia [18]. Nearly 50 years later, there are no data to support use of liothyronine to augment psychiatric medications in euthyroid children or adolescents.

Despite the potential psychiatric benefits reported in adults, administration of thyroid hormone to euthyroid patients carries the risk of inducing a hyperthyroid state. Some psychiatrists advocate for use of levothyroxine or liothyronine only in patients with depressive symptoms and TSH in the upper half of the reference range (above 2-2.5 *μ*IU/mL) because the risks of side effects, with the exceptions of atrial fibrillation and osteoporosis, are considered mild [17]. While some patients may accept the trade-off of the side effects of subclinical hyperthyroidism for the psychiatric benefits, there remains a lack of data on long-term cardiovascular and metabolic outcomes [5].

Safety considerations for liothyronine augmentation of psychiatric regimens include periodic laboratory monitoring of thyroid hormone levels, including total T3 [5, 6]. Studies recommend starting at a dose of liothyronine 25 *μ*g daily when used as an adjunct for depression with repeat of thyroid function tests at three months and then every six months [5, 6]. However, given that liothyronine has a substantially shorter half-life after oral administration than levothyroxine, single measurements may miss supraphysiologic peaks [19]. Due to the short half-life of liothyronine, clinical signs and symptoms of thyrotoxicosis after a dose increase may develop before detection by interval lab monitoring, as occurred in this case.

Awareness about availability of liothyronine is important because it may also be encountered in supplements and in compounded prescriptions. D'Arcy et al. described a case of exogenous T3 toxicosis in a man who took weight-loss supplements. He presented with complaints of palpitations and diaphoresis and exhibited tachycardia, hypertension, absence of goiter, and tremors. His thyroid-function studies, similar to those of our patient, showed low TSH, low free T4, and elevated free T3 [8]. Reports in the literature have also described compounding errors related to liothyronine in adults, including a report of 20-year-old male who received more than a thousand times the intended dose and had severe thyrotoxicosis symptoms and myocardial involvement with elevated troponin and ischemic changes [20].

## 4. Conclusion

Our case demonstrates the importance of both caution prior to prescribing liothyronine use in adolescents and consideration of exogenous liothyronine administration as a cause when patients present with symptoms of thyrotoxicosis with low TSH and low free T4. If liothyronine is prescribed, close monitoring should involve regular clinical assessment, laboratory measurement of thyroid function, and counseling about symptoms of thyrotoxicosis that can develop soon after liothyronine initiation or titration. If thyrotoxicosis develops, liothyronine should be discontinued or reduced to minimize adverse effects. As was seen in this case, thyroid function is expected to normalize following discontinuation of liothyronine.

## Figures and Tables

**Table 1:** Laboratory values before and after discontinuing liothyronine therapy.

Laboratory value (reference range)	At initial presentation	Subsequent work-up	3 months after discontinuation of liothyronine
TSH (0.34-5.60 *μ*IU/mL)	**0.02**		2.92
Free T4 (0.58-1.20 ng/dL)	**0.35**		0.76
Total T3 (87-188 ng/dL)		**466**	127
Sodium (136-144 mmol/L)	141		
Potassium (3.6-5.1 mmol/L)	4.2		
Calcium (8.5-10.1 mg/dL)	9.9		
C-reactive protein (0.0-10.0 mg/L)	<0.2		

## Data Availability

Data available upon request.

## Abbreviations

MDD: Major depressive disorder
SNRI: Serotonin-norepinepherine reuptake inhibitor
SSRI: Selective serotonin reuptake inhibitor
STEMI: ST-elevation myocardial infarction
TCA: Tricyclic antidepressant
TSH: Thyroid-stimulating hormone
T3: Triiodothyronine
T4: Thyroxine.
